# An Internet Tool for Creation of Cancer Survivorship Care Plans for Survivors and Health Care Providers: Design, Implementation, Use and User Satisfaction

**DOI:** 10.2196/jmir.1223

**Published:** 2009-09-04

**Authors:** Christine E Hill-Kayser, Carolyn Vachani, Margaret K Hampshire, Linda A Jacobs, James M Metz

**Affiliations:** ^2^Department of Medical OncologyUniversity of PennsylvaniaPhiladelphiaPAUSA; ^1^Department of Radiation OncologyUniversity of PennsylvaniaPhiladelphiaPAUSA

**Keywords:** Survivors, cancer survivor, patient care planning, survivorship care plan, late effects from cancer treatment, survivor issues, Internet

## Abstract

**Background:**

Survivorship care plans have been recommended by the Institute of Medicine for all cancer survivors. We implemented an Internet-based tool for creation of individualized survivorship care plans. To our knowledge, this is the first tool of this type to be designed and made publicly accessible.

**Objective:**

To investigate patterns of use and satisfaction with an Internet-based tool for creation of survivorship care plans.

**Methods:**

OncoLife, an Internet-based program for creation of survivorship care plans, was designed by a team of dedicated oncology nurses and physicians at the University of Pennsylvania. The program was designed to provide individualized, comprehensive health care recommendations to users responding to queries regarding demographics, diagnosis, and cancer treatments. After being piloted to test populations, OncoLife was made publicly accessible via Oncolink, a cancer information website based at the University of Pennsylvania which averages 3.9 million page views and over 385,000 unique visits per month. Data entered by anonymous public users was maintained and analyzed.

**Results:**

From May 2007 to November 2008, 3343 individuals utilized this tool. Most (63%) identified themselves as survivors, but also health care providers (25%) and friends/family of survivors (12%). Median age at diagnosis was 48 years (18 - 100+), and median current age 51 (19 - 100+). Most users were Caucasian (87%), female (71%), and college-educated (82%). Breast cancer was the most common diagnosis (46%), followed by hematologic (12%), gastrointestinal (11%), gynecologic (9%), and genitourinary (8%). Of all users, 84% had undergone surgery, 80% chemotherapy, and 60% radiotherapy. Half of users (53%) reported receiving follow-up care from only an oncologist, 13% only a primary care provider (PCP), and 32% both; 12% reported having received survivorship information previously. Over 90% of users, both survivors and health care providers, reported satisfaction levels of “good” to “excellent” using this tool.

**Conclusions:**

Based on our experience with implementation of what is, to our knowledge, the first Web-based program for creation of survivorship care plans, survivors and health care providers appear both willing to use this type of tool and satisfied with the information provided. Most users have never before received survivorship information. Future iterations will focus on expanding accessibility and improving understanding of the needs of cancer survivors in the era of the Internet.

## Introduction

Advances in cancer screening, detection, and treatment have increased the numbers of persons considered cured of cancer and those living with cancer as a chronic illness; as a result, the number of cancer survivors living in the United States (US) tripled from 3.0 million in 1971 to 9.8 million in 2001 [1]. A significant portion of the adult population is thus faced not only with the medical needs of normal aging, but with the unique health care concerns associated with cancer diagnosis and treatment, including recurrent and/or residual disease, treatment-related late effects, and threats to psychosocial and economic well-being [2-4].

Despite the unique needs of cancer survivors, this population may be at risk for receiving inadequate health care [5], and several groups have demonstrated that cancer survivors, a growing subset of the population, are at risk not only for cancer recurrence, but for receiving inadequate risk-based and routine preventive health care [5-7]. In response to this, national and international organizations have prioritized issues of survivorship over the past decade. In 2005, The Institute of Medicine (IOM) produced its report *From Cancer Patient to Cancer Survivor: Lost in Transition*. In this publication, the IOM outlined 10 recommendations intended to improve care of, and fiscal support for, cancer survivors. The second of these recommendations called on health care providers to provide patients with a “Survivorship Care Plan,” or “a comprehensive care summary and follow-up plan” [8]. This recommendation is based on recognition that many cancer survivors do not receive comprehensive care after active treatment, and that inadequate communication likely contributes to this. Indeed, the majority of primary care providers (PCPs) surveyed rate the current transition process from oncologic care to the PCP as fair or poor [9], and up to one-third of cancer survivors report being unsure of which of their physicians is in charge of their follow-up care [10]. Survivorship care plans are a conduit not only between active cancer care and survivorship care, but between physicians and survivors.

The Internet is an increasing source of health information worldwide. The Pew Internet & American Life Project reported in 2006 that 113 million Americans had used the Internet for health-related purposes; of these, over 50% reported that Internet use impacted their health care [11]. As the complexity of Internet-based systems has increased, several groups have demonstrated improvement in quality of life [12-13] and overall care [14-16] with use of Internet-based coaching for management of pain, diabetes, and heart/lung disease. Cancer is one of the top three diseases about which Internet users seek information [17], and recent studies suggest that the Internet may offer opportunities to actively improve health care for cancer patients and survivors [18]. Use of the Internet to actively manage symptoms related to cancer treatments is currently being examined in a European clinical trial [19].

In May 2007, we launched the world’s first Internet-based tool for creation of survivorship care plans, *OncoLife* [20]. *OncoLife* is a publicly accessible tool that is available through *OncoLink* [21], a cancer information website based at the University of Pennsylvania’s Abramson Cancer Center. *OncoLife* was designed to supply dynamic, personalized information to cancer survivors, and to prompt interventions with regard to both surveillance and management of late effects when indicated. The launch of *OncoLife* was anticipated to fill an unmet need for survivorship information and care; however, the willingness of survivors and their health care providers to use this type of tool, the satisfaction it would provide them, and the demographic, diagnosis, and treatment characteristics of users could not be predicted. The study described here was undertaken in order to investigate these questions. Our findings, as well as the *OncoLife* design and implementation process, are described here.

## Methods

**Figure 1:**
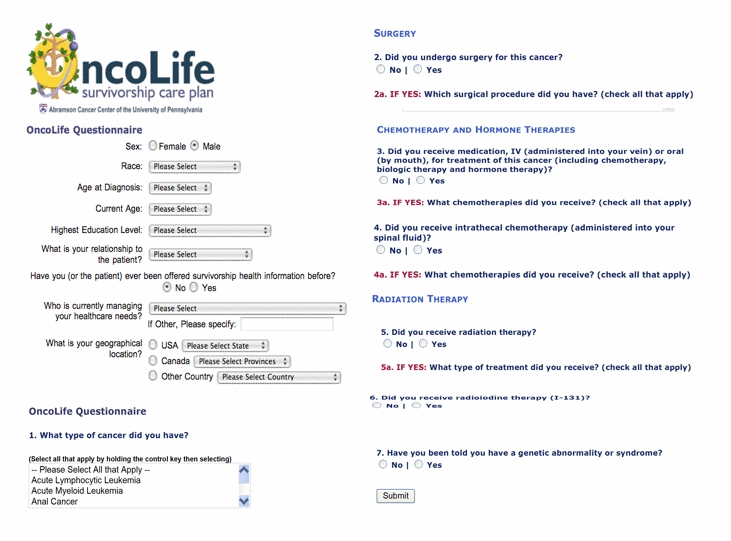
*OncoLife* user interface. The *OncoLife*  tool for creation of survivorship care plans is available via *OncoLink*  and *OncoLife*  websites

*OncoLink* is a general cancer information website maintained by physicians and nurses at the Abramson Cancer Center of the University of Pennsylvania, serving 3.9 million pages to over 385,000 unique Internet Protocol (IP) addresses monthly. *OncoLife*, a section of *OncoLink,* was developed by a dedicated team of oncology nurses and physicians. The *OncoLife* format includes a publicly accessible, five-screen series of 17 queries regarding demographics, cancer diagnosis, and cancer treatments received, and provides users with lists from which to select surgeries, sites of radiotherapy, and chemotherapy/biologic agents by both generic and trademark names (Figure 1).

The *OncoLife* survey is of open design, accessible to any visitor to the *OncoLink* site, with a target population of cancer survivors, health care providers, and friends/family members of survivors and a convenience sample frame. *OncoLife* is advertised via *OncoLink* (See Multimedia Appendix 1); flyers and bookmarks with the *OncoLife* trademark and website address are also available in our clinic and have been made available to health care providers in other institutions for distribution.

Completion of the *OncoLife* survey results in generation of individualized, detailed, comprehensive survivorship care plans providing surveillance recommendations for tumor recurrence, in addition to guidelines for overall health care in the setting of increased risk for certain morbidities secondary to cancer treatment (See Multimedia Appendix 2). These guidelines have been designed to be specific to types of treatments that patients have received, as well as their primary cancer diagnoses. Guidelines are based on type and site of surgical procedures, radiotherapy sites, and specific drugs received. All survivors are provided with information regarding second malignancy and other global issues pertaining to cancer survivorship. Guidelines are evidence- or consensus-based whenever possible and are in accordance with guidelines provided by the IOM, Children’s Oncology Group (COG), National Cancer Institute (NCI), and American Society of Clinical Oncology (ASCO). In areas in which evidence- or consensus-based guidelines are not available, guidelines provided are based on practice at our own institution. All guidelines provided as part of *OncoLife* survivorship care plans have been constructed with both nursing and physician input and are described in plain language.

The *OncoLife* survey and the information provided to survivors using *OncoLife* were piloted with groups of survivors who tested usability and technical functionality, prior to the public launch. The pilot version of *OncoLife* included queries regarding cancer diagnosis and treatment only (Table 1). Following the pilot process, queries regarding demographics were added, and version 1 was made publicly accessible. Over the course of the 18 months following the implementation of *OncoLife*, three further iterations were developed with the intent of increasing the comprehensive nature of the survivorship care plans produced by *OncoLife*, as well as increasing accessibility through improved understanding of the user population. Changes incorporated into iterations were based on user feedback, as well as observations regarding use patterns. Version 2 included additional queries regarding follow-up care and the availability of survivorship care and also requested that individuals completing the survey describe themselves as survivors, friends/family members of a survivor, or health care providers. Version 3 made use of the same series of queries used in version 2, but provided more individualized and extensive information to survivors, including adaptive questioning regarding surgeries, radiotherapy, and chemotherapies implemented for survivors specifically of breast cancer. Additionally, with the launch of version 3, a five-question, one-page user satisfaction survey was added through an optional link accessible upon receipt of survivorship care plans. Version 4, launched in January 2009, includes queries regarding menopausal status to allow further individualization of guidelines provided.

**Table 1:** *OncoLife* queries and response options according to version (vers.)

		Pilot Version	Vers. 1	Vers. 2^a^	Vers. 3^a^	Vers. 4
Number of Users (Non-duplicate)		40	1374	1124	805	Recent Launch
*OncoLife* Query	Response Options					
Sex	Male		•	•	•	•
	Female					
Race	Caucasian		•	•	•	•
	African American					
	Asian/Pacific Islander					
	Hispanic/Latino/a					
	Mixed Race					
	Other					
Age at Diagnosis	Please select		•	•	•	•
Current Age	Please select		•	•	•	•
Highest Education Level	Grade School		•	•	•	•
	High School					
	Some College					
	College Degree					
	Graduate School					
What is your relationship to the patient?	Self			•	•	•
	Family member/friend					
	Health care provider					
Have you ever been offered survivorship health information before?	Yes			•	•	•
	No					
Who is currently managing your health care needs?	Oncologist			•	•	•
	PCP/internist					
	Oncologist and PCP					
	Other (specify)					
What is your geographical location?	USA (select state)			•	•	•
	Canada (select province)					
	Other country (select)					
What type of cancer did you have?	Please select	•	•	•	•	•
Did you undergo surgery for this cancer?	Yes (select procedure)	•	•	•	•	•
	No					
Did you receive medication, intravenous or oral, for treatment of this cancer?	Yes (select medication[s])	•	•	•	•	•
	No					
Did you receive intrathecal chemotherapy?	Yes (select medication)	•	•	•	•	•
	No					
Did you receive radiation therapy?	Yes (select site)	•	•	•	•	•
	No					
Did you receive radioiodine therapy (I-131)?	Yes	•	•	•	•	•
	No					
Have you ever been told you have a genetic abnormality or syndrome?	Yes	•	•	•	•	•
	No					
What is your menopausal status? (females only)	Menopause before cancer therapy					•
	Postmenopausal (due to surgery or chemo/radiotherapy)					
	Premenopausal					
	Perimenopausal					
	Not sure					

^a^ Versions 2 and 3 differ only by survivorship care plans (SCP) generated.

In addition to its evolution through these versions, *OncoLife* has been completely translated into Spanish. Spanish translation was, and continues to be, performed by a bilingual (English- and Spanish-speaking) health care provider practicing in the field of oncology, with culturally relevant revisions occasionally made to the wording used on the website.

*OncoLife* remains an anonymous tool, and users are not asked for identifying information. Prior to submission of the *OncoLife* survey, users are able to review and change answers; however, in order to protect and ensure anonymity, users are not asked to “log in,” and entries are not maintained or saved for reuse or review at a later date. Use of *OncoLife* surveys is completely voluntary, with production of the survivorship care plan being the only incentive for use. Survivorship care plans produced using *OncoLife* were designed to address issues faced by adult cancer survivors. Pediatric cancer survivors are referred on the *OncoLife* introductory page to the COG website guidelines for survivors of childhood cancer [22]. *OncoLife* survivorship care plans are intended to provide guidance for survivors and physicians providing follow-up care to survivors, and they are not intended to replace interactions or recommendations provided by health care providers of individual survivors. Instead, plans may serve as aids for communication between survivors and their caregivers.

Data from each use of *OncoLife* have been maintained anonymously on a secure server, with automatic database entry. Data collection and maintenance procedures were approved by the Institutional Review Board (IRB) prior to the launch of *OncoLife*. Only data from completed questionnaires are recorded and/or analyzed—JavaScript encryption ensures that surveys cannot be submitted without completion of all queries. Where appropriate, queries provide non-response options (such as “I don’t know,” or “not applicable”). Data are password protected and are available only to the small team of physicians and nurses (five in total) involved in the creation of *OncoLife*. Entries are screened by IP address to avoid analysis of duplicate entries. A Chi-squared contingency table with one degree of freedom was used to compare user survey data regarding availability of information reported by survivors versus health care providers; an exact contingency test was used to compare satisfaction data after binning of Likert-type responses between the two groups to account for sparse cell population [23].

## Results

Between May 2007 and November 2008, 3647 *OncoLife* surveys were completed, 40 using a pilot version, 1562 using version 1, 1211 using version 2, and 834 using version 3. Based on duplicate IP address and data entry, 304 of these were identified as duplicates, leaving 3343 unique *OncoLife* users. Of these, 79 reported more than one cancer diagnosis. Of the 3343 responders, the median age at the time of cancer diagnosis was 48 years (mean 48, range 18 - 100+). Median current age was 51 years (mean 51, range 18 - 100+). The majority of users were women (71.3%, n = 2385) and described themselves as Caucasian (85.6%, n = 2861) and college-educated (78.2%, n = 2617) (Table 2). Of 1880 users who completed *OncoLife* surveys after the implementation of its second version, most described themselves as survivors (64.2%, n = 1198), although significant proportions were health care providers (24.8%, n = 461) and friends/family members of survivors (12.4%, n = 221) (Table 2). Health care providers were predominantly nurses (61.8%, n = 285) and nurse practitioners (23.2%, n = 107). Of 1872 users for whom data on location were available, the majority (91.0%, n = 1704) were US residents, representing 48 different states, and 5.9% (n = 110) were Canadian. The remaining 3% (n = 58) of users were residents of 24 other countries.

**Table 2:** Demographic information reported by users of *OncoLife*

Demographic		Total n = 3343	%
**Sex**			
	Male	957	28.6
	Female	2385	71.3
**Race**			
	Caucasian	2861	85.6
	African American	179	5.4
	Asian/Pacific Islander	91	3
	Hispanic/Latino/a	85	3
	Mixed Race	36	1
	Other	47	1
	Unknown	40	1
**Education**			
	Grade School	73	2
	High School	612	18.3
	Some College	699	20.9
	College Degree	1107	33.1
	Graduate School	811	24.3
	Unknown	40	1
**Relationship to Patient**		n = 1880^a^	
	Self	1198	64.2
	Family member/friend	221	12.4
	Health care provider	461	24.8
	Nurse	285	61.8^b^
	Nurse practitioner	107	23.2^b^
	Physician	48	10^b^
	Other health care	26	6^b^

^a^ Query added with implementation of version 2, so data available for n = 1880 users.

^b^ Refers to percent of health care providers (n = 461)

Breast cancer represented the most commonly reported primary cancer diagnosis (45.9%, n = 1537) among the 3343 *OncoLife* users, followed by hematologic (12.0%, n = 401), gastrointestinal (11.7%, n = 391), gynecologic (8.6%, n = 287), and genitourinary malignancies (8.3%, n = 278) (Table 3). Overall, 79.8% of these 3343 users (n = 2670) reported being treated with chemotherapy, 59.0% (n = 1973) with radiotherapy, and 83.5% (n = 2793) with surgery.

**Table 3:** Primary cancer diagnoses among users of *OncoLife*

Primary Cancer Diagnosis/Site	Number of *OncoLife* Users	%
Breast	1537	45.9
Hematologic	401	12.0
Gastrointestinal	391	11.7
Gynecologic	287	8.6
Genitourinary	278	8.3
Thoracic	149	4.5
Head & Neck	90	3
Melanoma	69	2
Central Nervous System	59	2
Thyroid	46	1
Sarcoma	38	1
Non-Melanoma Skin Cancer	17	< 1
Other	81	2

**Figure 2:**
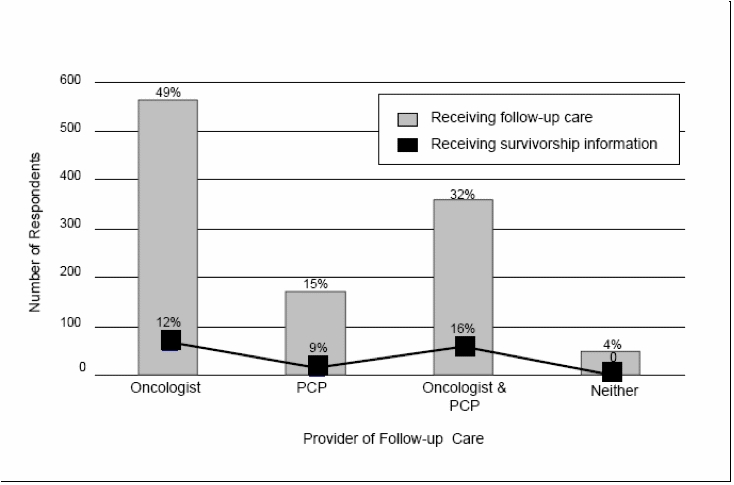
Follow-up care and survivorship information offered to users of *OncoLife*

Of 1869 users who provided information regarding follow-up care, half (52.5%, n = 982) reported receiving follow-up care only from an oncologist, and only 12.6% (n = 235) reported having previously received information on cancer survivorship (Figure 2). The majority of patients having received survivorship information prior to *OncoLife* were followed by an oncologist: Of these 235 patients, 89.7% (n = 211) reported receiving follow-up care from an oncologist, and 10% (n = 14) only from a PCP.

The user satisfaction survey was launched in July 2008, and 150 satisfaction surveys were completed. Of these, 57% (n = 86) were completed by survivors or family members/friends of survivors. The remaining 43% (n = 64) were completed by health care providers. According to overall user response, *OncoLife* survivorship care plan questionnaires took an average of 6.7 minutes to complete (range 1 - 30 minutes). Health care providers reported average time of 4.4 minutes, compared to 7.2 minutes reported by survivors and friends/family members. Overall, over 90% of users rated their experience and level of satisfaction using *OncoLife as* “good,” “very good,” or “excellent.” Specifically, 98% (n = 64) of the 65 health care providers rated their experiences as “good” to “excellent.” This was similar to 95% (n = 81) of the 86 survivors/friends/family members rating their experience “good” to “excellent” (probability = 0.2, *P* = .39). Most users (92%, n = 138) felt that they had the information needed to complete the *OncoLife* questionnaire, and this did not differ significantly between health care providers and survivors/friends/family members (89% [n = 57] versus 94% [n = 81] respectively [χ^2^ = 0.16 x 10^-3^, *P* = .99]). Most survivors (83%, n = 71) answered that they would plan to share the information provided with their health care team. Health care providers reported “good” to “excellent” levels of satisfaction with the information provided to the patient via *OncoLife* in 95% of cases (Table 4).

**Table 4:** *OncoLife* user satisfaction survey queries and responses

*OncoLife* User Satisfaction Survey Query	Response Options	Total Responses n = 150 (%)	Number Health Care Provider n = 64 (%)	Number Survivors/ Family Members/ Friends n = 86 (%)	*P*
How would you rate your experience completing this survey?	1 = Poor	0	0	0	.39^a^
	2 = Fair	5 (3)	1(2)	4 (5)	
	3 = Good	18 (12)	11 (17)	7 (8)	
	4 = Very good	43 (27)	16 (25)	27 (31)	
	5 = Excellent	84 (56)	36 (56)	48 (56)	
How long did it take to complete (in minutes) (Free entry)	Mean	6.7	4.4	7.2	n/a
	Median	5	5	5	
Did you have all of the information needed to complete the questionnaire?	Yes	138 (92)	57 (89)	81 (94)	.99
	No	12 (8)	7 (11)	5 (6)	
Was the information helpful?^b^	Yes		n/a	83 (97)	n/a
	No		n/a	3 (3)	
Will you share your plan with your health care team?^b^	Yes		n/a	71 (83)	n/a
	No		n/a	15 (17)	
How satisfied are you with the information provided to the patient?^c^	1 = Poor		0	n/a	n/a
	2 = Fair		3 (5)	n/a	
	3 = Good		17 (27)	n/a	
	4 = Very good		15 (23)	n/a	
	5 = Excellent		29 (45)	n/a	

^a ^For comparison of users responding “good” - “excellent” vs “fair” - “poor,” based on exact contingency test [23].

^b ^Query posed to survivors/family members/friends only

^c ^Query posed to health care providers only

## Discussion

Here we describe the design and implementation of, as well as use patterns and user satisfaction with, Internet-based survivorship care plans for cancer survivors. To our knowledge, *OncoLife* is the first such tool to be made publicly available. The intent of survivorship care plans is multifold: Plans are developed to assist with communication between physicians of various specialties, to increase physician-patient communication, and to increase awareness in both the physician and survivor populations of known and suspected late-effects associated with cancer and its treatments. The use of the Internet to allow creation of survivorship care plans allows information to be widely accessible and instantly available.

Based on the NCI definition of a cancer survivor, which includes all people diagnosed with cancer, as well as their caregivers, as survivors, several phases of survivorship certainly exist, and the needs of any one survivor may change dramatically over time. *OncoLife* survivorship care plans may be of use to survivors in any phase—from the moment of diagnosis until the end of life; however, their design may be most appropriate for those survivors who have completed cancer treatment or who continue to receive long-term cancer treatment. For this group of survivors, for whom the acute phase of cancer treatment may have ended, the designation of which health care provider(s) will provide various types of health care may be particularly ambiguous. This transition period may be associated with both survivor and physician uncertainty and dissatisfaction [9,10], potentially leading to important disparities in health care. Although all survivors may potentially be at risk for receiving inadequate health care after cancer treatment, prior data suggests those followed by an oncologic specialist may be more likely to receive adequate screening for late effects and disease recurrence [6], while those followed by a PCP may be more likely to receive adequate preventive care [5,7]. In reality, however, most survivors do not appear to be followed by both types of providers [7], a finding that is confirmed by users of *OncoLife:* Approximately one third of survivors using *OncoLif*e reported routinely receiving follow-up care from both a PCP and an oncologist. Of the remaining two-thirds, the majority reported seeing only an oncologist. These findings emphasize the need for comprehensive communication among physicians and between physicians and survivors. The vast majority of survivors utilizing *OncoLife* reported never having received survivorship information, and this suggests a broad communication deficit. Both PCP feedback and the improvement in comprehensive care when survivors are followed by multiple physicians indicate that gaps in communication are a significant barrier to care of cancer survivors. Survivorship care plans are a communication bridge between physicians and survivors, allowing all of the individuals involved in a survivor’s care (including the survivor) to be aware of survivorship health issues and to be assured that they are addressed.

According to the IOM, survivorship care plans should address issues of health maintenance, cancer screening, healthy behaviors, late effects of treatment, possible signs of recurrence, second malignancy risk, and financial consequences of cancer, and they should offer referrals to follow-up providers and lists of cancer-related resources [8]. Not surprisingly, in our current milieu of shrinking resources, the oncology community has expressed concern regarding time and monetary constraints limiting the feasibility of offering survivorship care plans, specifically voicing concerns that a survivorship care plan tool requiring more than 20 minutes per patient would be unrealistic [25]. The IOM recommended in its report that the service of provision of survivorship care plans “be reimbursed by third-party payers of health care” [8]. Hopefully, this concept will become reality in the future—the Comprehensive Cancer Care Improvement Act, currently under consideration in the US (HR. 1078/S. 2790), would allow Medicare reimbursement for oncologists to create survivorship care plans. In the meantime, *OncoLife* has been designed as a free service that does not rely on insurance re-imbursement, and *OncoLife* surveys take on average less than 7 minutes to complete. Both survivors and health care providers report high levels of satisfaction utilizing *OncoLife,* a tool that provides survivors with timely, comprehensive information that addresses the goals delineated by the IOM without insurance or payment delays.

The data presented here demonstrate that survivors, as well as their family members, friends, and health care providers, appear to be willing to use this type of tool. From our data, certain subsets of survivors appear more likely to use *OncoLife* than others—breast cancer survivors represent approximately one-quarter of adult cancer survivors living in the US today (22%) [1] and 45% of *OncoLife* users. This stands in contrast to prostate cancer survivors, who represent the second most prominent survivor population in the US (17%) [1] but only 6% of *OncoLife* users. The disproportionately low use of *OncoLife* by prostate cancer survivors is in all likelihood multifactorial and may have to do with decreased awareness of survivorship issues in this population when compared to the breast cancer survivor population. Another contributing factor may be the overall increased frequency of Internet use by women as opposed to men for health care needs [26-27]. Additionally, *OncoLife* users were predominantly Caucasian, well-educated, and young when compared to the overall survivor population. In 2001, persons over 65 years represented 61% of all cancer survivors [1], while the median age of *OncoLife* users was 51 years. These findings may reflect increased Internet access and level of comfort with Internet use among younger survivors, and are consistent with findings from other groups demonstrating increased Internet use in young, highly educated cancer survivors [28] and under representation of African Americans in online cancer support groups [29]. Since the initial development of *OncoLife,* efforts have been made to increase accessibility to underserved populations, including translation of *OncoLife* into Spanish, distribution of *OncoLife* materials at national meetings to health care providers for distribution to patients, and use of *OncoLife* by nurses at the University of Pennsylvania who complete surveys for patients when they complete cancer treatment. The vast majority of health care providers utilizing *OncoLife* are nurses, and oncology nurses represent a tremendous resource for provision of survivorship care plans to survivors with limited access to the Internet. Efforts are underway to raise awareness among nurses nationwide of the *OncoLife* tool. Efforts to further increase accessibility will continue with future iterations. Additionally, as more centers make use of computer-based data gathering by and for patients, we expect that availability of *OncoLife* to patients completing cancer treatment will continue to increase.

The anonymous nature of *OncoLife* has been maintained in order to protect user privacy and alleviate survivor fear of discrimination following cancer diagnosis; data obtained via *OncoLife* use is, however, limited by its anonymous nature. Data are strictly based on user responses and cannot be verified or validated. National efforts are ongoing to provide cancer survivors with comprehensive summaries of all cancer treatments received, which can then be entered directly into a tool such as *OncoLife*. Future versions of this program may be interfaced directly with electronic medical records to ensure accuracy of all data. Although nearly all users reported having access to the information needed to create a care plan using *OncoLife*, it is conceivable that other survivors might not utilize the tool because of limited access to information needed to complete the survey. Additionally, because users are not required to “log in,” plans are not currently saved on our system, although they may be printed and/or converted to electronic files for users themselves to save (both options are available at the time of survivorship care plan production). Future *OncoLife* iterations may be developed with a log in option, so that users may return to their own plans and update their information in order to received updated guidelines. Other limitations of *OncoLife* are associated with the current lack of evidence allowing construction of guidelines for follow-up care of patients after cancer. Our data suggest that most cancer survivors utilizing *OncoLife* have undergone multimodality treatment and are at risk for late effects; however, recognition of this risk may not translate into clear screening recommendations: Cardiac toxicity is recognized as a concern for survivors of breast cancer [30], but ASCO guidelines for screening for cardiac late effects do not exist due to “the lack of direct, high-quality evidence on the benefits and harms of [this] screening” [31]. In the development of *OncoLife*, we described published, evidence-based guidelines whenever possible, and lacking those, consensus-based guidelines. In situations in which these types of published guidelines are not available, *OncoLife* information is provided to increase survivor and physician awareness of late effects and their possible treatments. Only a small fraction of *OncoLife* users (12%) reported ever having received survivorship information in the past. Certainly, our hope is that the information provided by *OncoLife*, whether evidence-, consensus-, or practice-based, will be useful to survivors, especially in a setting in which most report having had very little information offered to them. Future efforts will focus on increasing the individualization of *OncoLife* survivorship care plans, as well as understanding of the survivorship population in efforts to expand use and accessibility.

## Multimedia Appendix 1

The *OncoLink* homepage, with display of the *OncoLife* announcement.

## Multimedia Appendix 2

Sample *OncoLife* survivorship care plan, developed for a 69-year-old woman with history of breast cancer at age 61, treated with lumpectomy/sentinel lymph node biopsy, adriamycin and cytoxan chemotherapies, breast conserving radiotherapy, and tamoxifen followed by an aromatase inhibitor.

## Abbreviations

ASCO: American Society of Clinical Oncology
COG: Children’s Oncology Group
IOM: Institute of Medicine
IP: Internet protocol
IRB: Institutional Review Board
NCI: National Cancer Institute
PCP: primary care provider
US: United States
