# Supplementary material for: An Internet Tool for Creation of Cancer Survivorship Care Plans for Survivors and Health Care Providers: Design, Implementation, Use and User Satisfaction
Source: J Med Internet Res. 2009 Sep 4;11(3):e39. doi: 10.2196/jmir.1223 (PMC2762859; doi:10.2196/jmir.1223)
Supplement: Supplementary file 1 [file jmir_v11i3e39_app1.pdf]

FEATURES

- OncoLife
- Nurses Notes
- OncoPilot
- OncoTips
- Abramson Cancer Center
- Continuing Education
- Newsletter
- Cancer Clinical Trials Matching

Onco Pilot
Navigating the Cancer Journey

ONCOLINK ART GALLERY

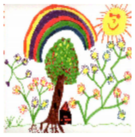

Today's artwork was donated by Olivia, a pediatric cancer patient who received treatment for cancer at The Children's Hospital of Philadelphia
Visit the Children's Art Gallery

CANCER NEWS

REUTERS

The latest news on cancer treatment, cancer research, oncology advances, and cancer clinical trials.

- Outcomes good with vulvar-conserving surgery even for large tumors [more]
- Retinoblastoma survivors often die from other malignancies [more]
- Longer hormone therapy may improve outcomes in advanced prostate cancer [more]
- Omacetaxine has potential to treat resistant leukemia [more]
- Marijuana use linked to nonseminoma testicular tumors [more]
- Autologous stem cell transplant useful in treatment of HIV-related lymphoma [more]

More Cancer News »

WHAT'S NEW

Ask The Experts
This is your chance to "Ask the Experts" everything you've always wanted to know about types of cancer, treatments and side effects.

- Cramping After BMT
- Mucinous Adenocarcinoma of the Prostate

Greetings from CancerLand: This is a test...
From breast cancer survivor and OncoLink's Poet-in-Residence, Alysa Cummings

Hürthle Cell Carcinoma
There are 4 main types of thyroid cancer, papillary (accounts for 75% of cases), follicular (15%), medullary (5%) and anapalstic (rare). Hürthle cell carcinoma is classified by the World Health Organization as a variant of follicular thyroid cancer, but others believe it is a distinctly separate disease.

Legal Words of Wisdom: COBRA
COBRA gives workers and their families who lose their health benefits the right to continue group health benefits provided by their group health plan for limited periods of time under certain circumstances.

Patient Art Gallery: Visitor Watercolor at NIH
From Artist David Beers

The 21st Century Cancer ALERT (Access to Life-Saving Early detection, Research and Treatment) Act

Legal Words of Wisdom: Financial Power of Attorney
From Rodney Warner, Esq, Legal Clinic for the Disabled, Inc.

Book Review: Choices in Breast Cancer Treatment
Alysa Cummings, OncoLink's Poet-in-Residence and breast cancer survivor, provides this book review.

View All What's New | What's New RSS

HOT SPOT

OncoLife
survivorship care plan
Abramson Cancer Center of the University of Pennsylvania

OncoLink has created an individualized plan of care based on the Institute of Medicine recommendations for cancer survivors. This free and easy to use program provides cancer survivors with information regarding the health risks they face as a result of cancer therapies. It encourages them to review the plan with their healthcare team to further assess their risk and become active participants in their follow up care.

Develop your survivorship care plan today!

Oncolife en españõl

CANCER AWARENESS

Minority Cancer Awareness Week
April 20-26

April 20-26, 2008 is Minority Cancer Awareness week

This annual event drawing attention to the disproportionate impact cancer has on minorities. Minority populations are more likely to be diagnosed with certain types of cancer and more likely to die from the disease. Read More...

FEATURED SECTIONS

Smoking Cessation Aids
This article will review the available treatments, both pharmacologic (drug) and non-pharmacologic, to aid in successful smoking cessation.

Smoking Cessation: Where do I Start?
The US Surgeon General stated that quitting smoking is the single most important step that smokers can take to enhance the length and quality of their lives.

Did You Know...The Facts About Smoking and the Worldwide Crisis it has Caused?
Smoking is the number one cause of lung cancer.

EXPERTS ON CALL

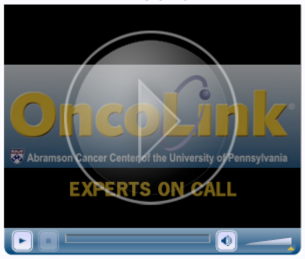

Dr. Anil Vachani discusses lung cancer prevention and early detection

Tune in to watch Dr. Anil Vachani, Assistant Professor at the University of Pennsylvania Medical Center discuss lung cancer risk and prevention, early detection of lung cancer, and smoking cessation including a new medication option.

Keep up-to-date with the latest cancer Information
OncoLink
Abramson Cancer Center of the University of Pennsylvania
www.oncolink.upenn.edu

Onco Link Art Gallery

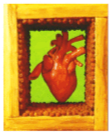

Art made by cancer patients and their loved ones to encourage vision, hope and imagination

Onco Pilot
Navigating the Cancer Journey
A guide developed to give suggestions on ways to make the time of diagnosis easier and to help one make the best decisions.
OncoPilot Navigating the Journey

Copyright © 1994-2009 Trustees of the University of Pennsylvania
