# Supplementary material for: An Internet Tool for Creation of Cancer Survivorship Care Plans for Survivors and Health Care Providers: Design, Implementation, Use and User Satisfaction
Source: J Med Internet Res. 2009 Sep 4;11(3):e39. doi: 10.2196/jmir.1223 (PMC2762859; doi:10.2196/jmir.1223)
Supplement: Supplementary file 2 [file jmir_v11i3e39_app2.pdf]

OncoLife survivorship care plan
Abramson Cancer Center of the University of Pennsylvania

OncoLife Results
Welcome to your personalized OncoLife plan of care. This plan is designed for survivors of adult cancers and was developed based on the information you entered on the questionnaire. Childhood cancer survivors are encouraged to visit the Children's Oncology Group website for more information specific to them. While every aspect of survivorship is important, including psychosocial, emotional and financial issues, this plan focuses on the long term medical risks the survivor may face.

The information in this plan is based on the available research and literature concerning cancer survivors. This area is continually growing and as new information becomes available, it will be added to the program. For this reason, you may want to redo your plan periodically. The goal of the OncoLife program is to provide cancer survivors with information regarding the health risks they face as a result of cancer therapies. The level of risk can vary based on the duration, doses and combinations of therapy, therefore this plan should be discussed with your oncology team to better understand your personal risks. These results can be frightening, but remember, not every survivor experiences every side effect, and some do not experience any long term effects. This plan is to make you aware of possible long term effects that you and your healthcare team should keep in mind.

The following sections are broken down by the cancer therapies you entered on the questionnaire. Sometimes more than one therapy can cause the same long term effect, so you may see a particular side effect in more than one section. If there are specific things you can do to reduce the risk for or evaluate for the presence of an effect, these will be summarized at the end of the section.

Your Summary

You were treated for breast cancer:

- lumpectomy
- sentinel node biopsy
- cyclophosphamide (cytoxan®, neosar®)
- doxorubicin (adriamycin®, rubex®, doxil®)
- exemestane (aromasin®)
- tamoxifen (nolvadex®)
- radiation treatment for breast cancer after lumpectomy

All Survivors

Coordinating Your Care

As a survivor, it is important that you keep a journal or notebook of your care. Include your doctor's contact information, medications taken, therapies received and radiology testing you have had. (Visit

the OncoPilot section for forms you can use to organize this material). While some survivors continue to see an oncologist, many return to a primary care provider or internist for care, many of whom are uncertain how to care for you. Developing the OncoLife plan of care can help you and your primary care provider in understanding what effects to look for and how to handle them. If you are being seen only by a primary care practitioner, it is a good idea to be known to an oncologist or late effects clinic, should you need any guidance or referrals with regards to late effects. The Cancer Survivors Project maintains a list of late effects clinics, which will review the therapies you received, discuss risks with you and act as a consultant to your primary care team.

Risk of a second cancer

As a survivor, your chance of developing a second cancer is about twice that of a person of the same sex and age who has never had cancer. This may be a different type of cancer altogether, or a cancer in the same site as before, that is not related to the first cancer. While this sounds scary, it is important to be aware of this risk and be proactive in your own healthcare. It is not well understood why survivors have this risk, but having follow up care, cancer screening and a healthy lifestyle can decrease your risk. In some cases, a treatment (types of chemotherapy or radiation therapy) increases the risk of another cancer. These are called secondary cancers because they develop as a result of therapy. If you are at risk for a secondary cancer, it will be discussed further in your plan.

Because of this risk, survivors are encouraged to adapt a healthy lifestyle of exercise, avoidance of tobacco use and alcohol only in moderation (less than two drinks a day for men and one for women), maintaining a healthy weight and eating a health conscious diet, including lots of fruits and vegetables. The American Institute for Cancer Research has developed nutrition guidelines for cancer survivors to address questions related to diet. Practice safe sun habits by using sunscreen, wearing protective clothing and not using tanning booths. Survivors should follow recommended guidelines for cancer screening, with earlier screening if they are in a high risk category (i.e. radiation to an area, genetic syndrome).

The following sections will address risks related to the therapies you received.

Fatigue

Fatigue is the most common side effect of cancer treatment. What many people do not know is that this feeling of overwhelming physical, mental and emotional exhaustion can last for months to years after therapy ends. Soon after treatment is complete, friends, family and co-workers often expect the survivor to be back to doing the things they did before treatment, with the same vigor. Many survivors report significant fatigue years after completing therapy, which can be extremely frustrating for the survivor and those around them. There have been many studies examining fatigue and ways to combat it during treatment, but there is little to provide guidance for dealing with fatigue after therapy. It is important to remember that fatigue can be caused by many things and, particularly if fatigue is worsening or new, it should be discussed with your healthcare team to rule out treatable causes.

Research has shown that light exercise can aid in relieving fatigue during treatment, so it is possible that this could help post treatment. Talking with other survivors may help in finding ways to deal with fatigue. Most importantly, you should understand it is normal and you will need to give your body time to slowly return to your former energy levels. With a lack of available interventions proven to relieve fatigue, survivors may need to learn to work around it in a sense. A wise survivor once dubbed herself the "master of fatigue" because, she stated, "I had learned how to outsmart it". By thinking of her energy as a full bowl of candy each morning, and each task a certain number of candies. She only had so many candies each day, so tasks needed to be prioritized and balanced with the amount of candies left in her bowl. By learning to manage tasks, group errands, make lists, prioritize and delegate, you

can, to an extent, outsmart your fatigue.

Risks Related to Medications

Risk of Developing Bladder Cancer

Cyclophosphamide and streptozocin can contribute to the development of bladder cancer. This risk is increased for those who also received radiation therapy to the abdomen. Symptoms of bladder cancer include blood in the urine, urinary frequency and urgency, urinating at night and incontinence and should be reported to the healthcare provider. Survivors should be counseled that alcohol use and smoking can contribute to bladder cancer, so these should be avoided.

Summary

- Avoid alcohol
- Avoid smoking
- Report the following symptoms to your healthcare provider
  - Pain when urinating
  - Urinary hesitancy – starting and stopping while urinating
  - Urinating frequently
    - Urinating more than 5 times per day
    - Getting up in the middle of the night to urinate
  - Blood in your urine

Risk of Bladder or Urinary Tract Toxicities

The risk for bladder and urinary track toxicities is highest for survivors who received cyclophosphamide (doses > $3g/m^2$), ifosfamide and/or radiation to the abdomen. Late effects to the urinary tract can include hemorrhagic cystitis, a condition characterized by bleeding from the bladder lining and bladder scarring leading to a decrease in the bladder capacity. Symptoms of hemorrhagic cystitis include urinary frequency and urgency, blood in the urine and pain. Bladder scarring can present as difficulty urinating, frequency or urgency. Survivors at risk should report these symptoms to their healthcare provider right away. Survivors should be counseled that alcohol use and smoking can contribute to bladder dysfunction, so these should be avoided.

Summary

- Avoid alcohol
- Avoid smoking
- Report the following symptoms to your healthcare provider
  - Pain when urinating
  - Urinary hesitancy (difficulty starting the stream)
  - Urinating frequently
    - Urinating more than 5 times per day
    - Getting up in the middle of the night to urinate
  - Blood in your urine

Risk of Developing Osteoporosis

Osteoporosis and osteopenia (the precursor to osteoporosis) are decreases in bone density, which increases the risk of fracture of the affected bones. Long term use of corticosteroids (dexamethasone or prednisone, > 5mg per day for more than 2 months), receiving chemotherapy medications (including methotrexate, ifosfamide, cytoxan) or radiation to weight bearing bones (spine, hips, legs) all increase the risk of developing osteoporosis.

Women who develop premature menopause, have their ovaries removed before menopause or those who take aromatase inhibitors (anastrozole, letrozole and exemestane) are at increased risk. Men who receive hormone therapy for prostate cancer or undergo orchiectomy are at risk. In addition, patients who have undergone gastrectomy (removal of the stomach) are at increased risk.

As for lifestyle risks, smokers, people who consume excessive alcohol and those who do not participate in weight bearing exercise have an increased risk of developing osteoporosis. Survivors at risk should have adequate intake of calcium (about 1200mg per day) and vitamin D (400-800 international units per day), participate in weight bearing exercise (walking, dancing, jogging or any exercise where the legs are supporting the body's weight) and talk to their healthcare provider about screening with DEXA scan and options for treatment, if necessary.

Summary

- Avoid smoking and excessive alcohol intake
- Perform weight bearing exercise 2-3 times per week
- Calcium intake of 1200mg per day plus Vitamin D 400-800iu per day (either in dietary intake or supplements)
- Consider screening with DEXA scan

Risk for Cardiac (Heart) Problems Related to Anthracycline Chemotherapies

The group of chemotherapy agents called anthracycline antibiotics are known to cause specific cardiac toxicities, including cardiomyopathy (weakening of the heart muscle), arrhythmias (rhythm abnormalities) and left ventricle dysfunction (causing heart failure). The risk of developing one of these problems is tied to the cumulative (lifetime) dose a person has received, but even low doses can lead to abnormalities. Toxicity can develop anywhere from shortly after completing chemotherapy (called chronic) to decades later (called delayed). For example, it is known that cumulative doses of doxorubicin greater than 550mg/m^2 can lead to cardiac toxicity, but doses as low as 250mg/m^2 can result in subclinical cardiac changes. Subclinical changes can be detected on tests such as ECG, echocardiogram and/or MUGA scan, but they do not cause symptoms for the survivor. The doses of the various anthracycline agents are not equivalent, so you should discuss the dose you received and your risk with your physician.

Risk is further increased for those survivors who also received radiation to the chest or those who received high dose cyclophosphamide (dose levels used in bone marrow and stem cell transplant preparation). Survivors should maintain healthy lifestyles as smoking, drug use, obesity, sedentary lifestyle and poor dietary choices can increase the risk of cardiac disease. Females who wish to become pregnant should be evaluated by a cardiologist prior to pregnancy and be followed by a high risk obstetrician.

Cardiac toxicities can cause symptoms such as shortness of breath (with or without exertion), orthopnea (difficulty breathing when lying down), chest pain, palpitations, exercise intolerance, dizziness/lightheadedness or edema (swelling of the extremities). In younger survivors (under age 25),

cardiac symptoms may present as abdominal symptoms such as nausea and vomiting. Annual history and physical by a healthcare provider should include a cardiac exam and review of possible symptoms. Survivors who received anthracyclines (any dose) should have their left ventricular function evaluated at baseline with an echocardiogram or MUGA (nuclear imaging radiology exam of heart function) scan, as studies have found many people with abnormalities did not exhibit symptoms. Repeat evaluation should be performed periodically (more frequently for higher risk individuals) or if symptoms develop or worsen.

Summary

- Maintain healthy lifestyle
  - Avoid smoking
  - Avoid drug use
  - Maintain a healthy weight
  - Exercise regularly Eat a well-balanced diet
- Women should have a cardiac exam by a cardiologist prior to becoming pregnant and be followed by a high risk obstetrician
- Have an annual physical exam that includes a cardiac exam
  - Periodic repeated cardiac studies (echocardiogram or MUGA)
- Report the following symptoms to your healthcare provider
  - Shortness of breath (with or without exertion)
  - Difficulty breathing when lying down
  - Chest pain / heartburn
  - Palpitations
  - Dizziness/lightheadedness
  - Swelling of the arms or legs
- If your received chemotherapy under age 25
  - Report symptoms of nausea and vomiting

Risk of Developing Cataracts

The risk of developing cataracts is linked to busulfan, corticosteroids (dexamethasone, prednisone), tamoxifen, anastrozole and radiation therapy involving the eye (including total body irradiation). Survivors should report any symptoms of cataracts and have an eye exam performed by an ophthalmologist every few years. Symptoms of cataracts include: blurry vision, light sensitivity, poor night vision, double vision in one eye, seeing halos around objects, needing brighter light to read or fading or yellowing of colors.

Summary

- Have an eye exam by an ophthalmologist every 1-2 years
- See your doctor immediately if you experience any cataract symptoms
  - Blurry vision
  - Light sensitivity
  - Poor night vision
  - Double vision in one eye
  - Seeing halos around objects
  - Needing brighter light to read
  - Fading or yellowing of colors

Risk of Liver Toxicity

Hepatic dysfunction is abnormal functioning of the liver. This can range from having abnormal results on a blood test with no symptoms to cirrhosis or liver failure. The majority of complications tend to occur during or soon after therapy and patients who receive methotrexate, mercaptopurine, thioguanine, BCNU (carmustine), plicamycin and tamoxifen are at highest risk of developing liver problems. Toxicities may resolve over time, but in some cases, can result in chronic liver problems.

The Risk of Developing a Blood Cancer

Certain chemotherapy medications can cause damage to the blood cells in the bone marrow. This damage can cause leukemia or myelodysplasia (MDS) to develop years after therapy has been completed. Both diseases cause an abnormal production of poorly functioning blood cells, making it difficult for the body to fight infection, carry oxygen to the tissues and prevent bleeding. Because these condition develop as a result of chemotherapy or radiation exposure, they are often more difficult to treat than typical leukemia or MDS.

Leukemia and MDS caused by chemotherapy or radiation therapy typically occurs between 4-10 years after treatment, but can occur even later. One exception is those caused by etoposide (VP-16) or teniposide (two types of chemotherapy), which generally occur within 1-3 years after therapy. Secondary lymphomas have also been seen in Hodgkin's disease survivors who received the MOPP (nitrogen mustard [mustargen], vincristine [oncovin], procarbazine, and prednisone) chemotherapy regimen.

Sexuality Concerns for Female Survivors

Women of any age may have sexuality concerns after cancer treatment. Do not hesitate to talk with your oncology team about these common concerns. Chemotherapy agents are associated with vaginal dryness, painful intercourse, reduced sexual desire and ability to achieve orgasm. Many of these issues are caused by the sudden onset of menopause, which can occur with cancer therapy. This sudden change in hormone levels leads to physical changes such as vaginal atrophy (thinning and inflammation of the vaginal walls), loss of tissue elasticity and decreased vaginal lubrication. In addition, women may experience hot flashes, mood swings, fatigue and irritability.

Decreased lubrication leading to painful intercourse is a common concern for survivors. This can often be treated with vaginal lubricants and moisturizers and/or estrogen therapy (taken orally or used in the vagina). Women who have had a hormone dependent cancer should discuss current research on using these therapies with their healthcare team. Surgery and/or radiation therapy can result in scarring that may cause discomfort during intercourse. Open communication about position changes and alternative methods of expressing affection with your partner can help when resuming sexual activity after treatment.

Concerns about changes in your body, cancer recurrence, the stress and anxiety caused by cancer therapy or changes in your relationship with your partner can all effect how you feel about your sexuality. It is important to understand that sexual activity cannot cause cancer to recur, nor can you spread cancer to another person through sexual activity. If you find that your feelings are significantly

impacting your sexuality, you should talk with your healthcare team about finding a therapist experienced in helping cancer survivors.

Of utmost importance in addressing sexuality issues is communication, both between partners and between survivors and their healthcare teams. Understand that these concerns are common and communication is the first step to finding the right solutions. Visit OncoLink's section on sexuality for more information.

Side Effects while taking Aromatase Inhibitors

Your OncoLife plan focuses on late effects of therapy, or those that can occur months to years after completing therapy. Current hormone therapy regimens last anywhere from 5 to 10 years, so we felt it was important to include some information about the acute side effects of these agents.

Aromatase Inhibitors (AIs) commonly cause hot flashes and other symptoms of menopause. Avoiding triggers such as warm rooms, spicy, caffeinated or alcohol containing foods or beverages can help reduce hot flashes. Drink plenty of fluids, wear breathable clothing and exercise regularly. For some women, certain antidepressant medications can provide relief of hot flashes.

Many women taking AIs experience aching in their muscles, joints or bones, also known as arthralgias. In some cases, this side effect is troubling enough for the patient to stop therapy. The cause of this pain is not clear, but it may be related to the low estrogen levels while on these medications. Arthralgias may occur in as many as 60% of women taking AIs. Some patients experience pain that comes and goes, in others it is constant and some report noting worse stiffness and pain in the morning.

Treatments commonly used are acetaminophen, nonsteroidal anti-inflammatory drugs (NSAIDS, ibuprofen, naproxen), narcotic pain relievers, glucosamine and topical pain relieving ointments. Research studies have used vitamin D therapy or acupuncture with some success. Further research is needed to determine the best therapies to manage this common side effect.

Side Effects While taking Tamoxifen

Your OncoLife plan focuses on late effects of therapy, or those that can occur months to years after completing therapy. Current hormone therapy regimens last anywhere from 5 to 10 years, so we felt it was important to include some information about the acute side effects of these agents.

Tamoxifen commonly causes hot flashes and other symptoms of menopause. Avoiding triggers such as warm rooms, spicy, caffeinated or alcohol containing foods or beverages can help reduce hot flashes. Drink plenty of fluids, wear breathable clothing and exercise regularly. For some women, certain antidepressant medications can provide relief of hot flashes.

The more serious, though low risk, complications of tamoxifen include endometrial cancer and blood clots. Women should promptly report any menstrual irregularities, vaginal bleeding, pelvic pressure/pain, or any vaginal discharge, as these may be symptoms of endometrial cancer. An endometrial biopsy should be done to test for cancer if any of these symptoms occur.

Blood clots are rare, but most often occur in the calf or lung. Signs of a blood clot in the leg may include any of the following: leg pain, warmth, swelling of one leg more than the other. Signs of a blood clot in the lung could include: fever, shortness of breath that comes on you very quickly, racing

heart, chest pain (that tends to be worse when you take a deep breath). Any of these symptoms should be reported to your physician immediately.

Skin Toxicities

Some chemotherapy agents will cause the skin to darken or lighten in spots or cause the nails to change color or fall off. While this typically happens while on therapy, these effects can become chronic. Good hygiene and skin care, including washing with a mild soap and water, hydrating lotions for dry or scaly skin, and protecting any open skin wounds can all aid in recovery from these toxicities.

Understanding "Chemo-brain"

Many survivors who have previously received or are currently undergoing chemotherapy report experiencing cognitive changes, often referred to as "chemo-brain." These changes include difficulty with short term memory, multi-tasking, new learning, reading comprehension, working with numbers and a decrease in concentration ability. For many years this was attributed, by physicians and researchers, to depression or anxiety over the diagnosis and treatment of cancer. More recently, researchers have begun to study and document what survivors have been saying all long; cognitive changes after chemotherapy are real. Although we are not yet able to pinpoint whether only certain chemotherapies are responsible, it seems certain that the effects are cumulative. That is, those who receive more chemotherapy tend to experience greater deficits. Studies have found that cognitive ability can improve over time in some survivors, but deficits are still present in many long term survivors, years after treatment.

Some medications are being studied as potential treatments for cognitive changes, but there is not yet enough data to support their use. Some of the agents being studied include: methylphenidate (Ritalin), modafinil (a medication approved to treat narcolepsy), various antidepressants, herbal therapies, such as ginkgo biloba, ginseng and certain amino acids. Cognitive rehabilitation programs are structured programs utilizing exercise, tasks that use memory and puzzles to "rehabilitate" one's mind. These programs are typically used for people with brain injuries, but therapists have tailored programs for cancer survivors. Bookstores and websites offer memory training, which may be helpful to survivors. Puzzles using numbers, like Sodoku, may help "exercise" your brain. Fatigue can enhance cognitive problems, so avoiding fatigue by getting enough sleep, incorporating exercise into your life and eating a healthy diet may be helpful.

It is important to remember that some very treatable problems can result in cognitive difficulties, such as thyroid dysfunction, depression and anxiety, so it is important to exclude or treat these diagnoses. Hypothyroidism (low thyroid hormone levels) is a common issue for survivors and can make you feel "fuzzy" or "out of it." This is easily treatable with supplemental thyroid hormone. Survivors who may be depressed or experiencing anxiety would benefit from consulting with a psychiatrist or psychologist experienced in working with cancer patients or survivors.

Surgery Side Effects

Lumpectomy

Surgery for breast cancer can include mastectomy (removal of the entire breast) or lumpectomy (sometimes called breast conserving surgery). These surgeries can result in cosmetic deformities. In some cases, these can be corrected with breast reconstruction performed by a plastic surgeon. There is a risk of nerve damage during breast surgeries, which could lead to pain in the chest wall or the arm/hand on the side of the surgery. Injuries like this could be aggravated by scar tissue formation after radiation therapy to the area. Survivors who develop chronic pain may benefit from a consult with a pain specialist.

Sentinel Node Biopsy

The removal of lymph nodes from the axillary (underarm), abdominal, or groin areas can lead to decreased drainage in the closest limb, causing lymphedema (a swelling of the limb) to result. Survivors who have also received radiation therapy to the area are at greater risk of developing lymphedema, which can occur years after therapy. Sentinel node biopsy typically removes only 1-3 lymph nodes to look for cancer cells. While this decreases the risk of developing subsequent lymphedema, the risk is not completely eliminated. Lymphedema can cause pain, disfigurement, functional limitations and increase the risk of a serious infection in that limb. A Certified Lymphedema Therapist should be consulted at the first sign of swelling to achieve the best outcomes. Survivors should be aware of this potential complication, given information on self-care and instructed to notify the healthcare team with any signs of swelling or infection.

Surgery to remove the lymph nodes (or sentinel node) can result in injury to the nerves in that area. Nerve damage can cause pain, numbness, tingling, decreased sensation or strength in the area or limb. For instance, axillary (underarm) lymph node removal can result in these symptoms on the chest wall, under the arm (armpit) or in the arm and/or hand on the treated side. Injuries like this could be aggravated by scar tissue formation after radiation therapy to the area. This type of pain is called nerve pain and is treated with different medications than other types of pain. Survivors with this type of pain may benefit from a consult with a pain specialist.

Radiation Side Effects

Long term effects of radiation therapy vary greatly depending on the areas included in the field of radiation and the radiation techniques that were used, as these continue to develop and improve. One issue that is consistent across all tissues is the possibility of developing a second cancer in or near the radiation field. Secondary cancers develop as a result of the exposure of healthy tissue to radiation. Newer radiation techniques are designed to limit this exposure, but it is not always possible to prevent all exposure and still achieve the desired outcomes.

Heart / Cardiovascular

Potential late effects of radiation fields including the heart include premature coronary artery disease and hypertension (high blood pressure), valve abnormalities, fibrosis or scarring of the cardiac tissue resulting in decreased heart function, pericarditis (inflammation of the heart sack), heart failure and myocardial infarction (heart attack). The actual risk varies greatly depending on the total dose of

radiation, number of fractions (doses), amount of radiation actually delivered to the heart, time since radiation and whether or not chemotherapy agents with known cardiac toxicity were also given. Survivors at risk should have a yearly history and physical by a healthcare provider to evaluate cardiac function and blood pressure and should be counseled on lifestyle choices including exercise, tobacco avoidance and a healthy diet. High risk survivors may benefit from yearly EKG and screening echocardiogram to evaluate heart function.

Survivors should also be especially aware of other factors that increase risk of heart disease and death from heart attack. These include high cholesterol, obesity, high blood pressure, diabetes, smoking, and illegal drug use (cocaine). Screening for all of these risk factors can allow for early intervention.

- Avoidance of tobacco and illegal drug use
- Yearly history and physical exam with monitoring of cholesterol levels, blood pressure, and blood sugar by primary care physician to reduce risk of heart disease/ attack
- Yearly EKG and/ or echocardiogram for high-risk patients

Lung

Radiation fields involving the lung can lead to scarring (fibrosis), inflammation (pneumonitis), and restrictive or obstructive lung disease. Risk for these problems is increased with higher doses of radiation and radiation given in combination with certain chemotherapies (bleomycin, busulfan, BCNU and CCNU) and for those survivors who also had part of the lung surgically removed (lobectomy). Survivors who have had radiation to the lung are strongly encouraged not to smoke, as this can greatly increase the risk of problems. Annual history and physical by a healthcare provider should include a pulmonary exam and review of possible symptoms (cough, shortness of breath, wheezing). Survivors should receive annual flu vaccines and the pneumococcal vaccine. Physicians may consider chest x-rays or pulmonary function tests for those at highest risk or a change in pulmonary status.

Scarring within the lungs can result from radiation, and uncommonly this scarring may affect blood vessels. Any survivor coughing up blood should be evaluated immediately by a physician, either in the office or the emergency room.

Of note, the Children's Oncology Group recommends survivors not scuba dive without medical clearance from a diving medicine specialist.

- Annual influenza vaccine
- Pnuemococcal vaccine every 5 years
- Tobacco avoidance/ smoking cessation
- Chest X-ray for new cough or shortness of breath
- Immediate evaluation of hemoptysis (coughing up blood)

Bone

Damage to the bone from radiation can cause small cracks (fractures) in that bone. The ribs are more susceptible to fracture after radiation, although these fractures will almost always heal normally. If radiation is given in the area of a joint, permanent stiffness, pain and arthritis can develop in that joint.

- Rapid evaluation for fractures after trauma (for example, after a motor vehicle accident)
- Physical/ Occupational therapy for arthritis
- Non-steroidal inflammatory medicines for arthritis

Skin

Radiation can lead to permanent changes in the skin. This can include changes in the color or texture of the skin, scars, and changes in the color, texture of hair or permanent loss of the hair in the treated area. The soft tissue and muscles under the skin can develop scarring and/or shrinkage, which can lead to a loss of flexibility and movement or chronic swelling. Some patients develop chronic or recurring ulcers of the skin in the area treated. Blood vessels of this skin may become dilated and more noticeable, although this is not harmful. If the skin feels tight or sore, regular use of vitamin E applied to the skin can be helpful.

After radiation the skin is more sensitive to sunlight, and survivors should be especially cautious to use sunscreens when outdoors.

- Diligent use of sunscreen
- Evaluation by a wound care specialist or surgeon for non-healing ulcers

Lymph nodes

The removal of lymph nodes from the axillary (underarm), abdominal, or groin areas can lead to decreased drainage in the closest limb, causing lymphedema (a swelling of the limb) to result. Survivors who have also received radiation therapy to the area are at greater risk of developing lymphedema, which can occur years after therapy. While sentinel node biopsy can decrease the risk of developing subsequent lymphedema, the risk is not completely eliminated. Lymphedema can cause pain, disfigurement, functional limitations and increase the risk of a serious infection in that limb. A Certified Lymphedema Therapist should be consulted at the first sign of swelling to achieve the best outcomes. Survivors should be aware of this potential complication, given information on self-care and instructed to notify the healthcare team with any signs of swelling or infection.

Surgery to remove the lymph nodes (or sentinel node) can result in injury to the nerves in that area. Nerve damage can cause pain, numbness, tingling, decreased sensation or strength in the area or limb. For instance, axillary (underarm) lymph node removal can result in these symptoms on the chest wall, under the arm (armpit) or in the arm and/or hand on the treated side. Injuries like this could be aggravated by scar tissue formation after radiation therapy to the area. This type of pain is called nerve pain and is treated with different medications than other types of pain. Survivors with this type of pain may benefit from a consult with a pain specialist.

Radiation for breast cancer (after lumpectomy)

Any patient who has had breast cancer is at risk for developing a second breast cancer in either the treated or the opposite breast. It is recommended that women undergo annual mammograms after treatment for breast cancer, as well as yearly breast exams by a breast cancer specialist (medical oncologist, radiation oncologist, or breast surgeon).

Some women who receive radiation to the breast may later want to breastfeed. It is possible for the irradiated breast to produce small amounts of milk, but it is not recommended to use this breast for feeding because of the risk of mastitis (inflammation/infection of the breast tissue), which could be difficult to treat. If only one breast was treated, the untreated breast should produce milk and can be used safely for breastfeeding.

Other long term effects of breast irradiation include changes in the size or shape of the breast and damage to the nerves, leading to pain or loss of strength or feeling in the arm on the side that was

irradiated. Damage to the drainage (lymphatic) system in the area can lead to chronic swelling, called lymphedema. Risk of lymphedema is highest for women who also had surgical lymph node dissections and, to a lesser extent, sentinel node biopsy. A survivor with lymphedema who develops pain or redness in the arm, especially with fever, should be evaluated as these signs may indicate infection.

Survivors of breast cancers, particularly left-sided breast cancers, may be at increased risk of cardiac complications. Please see the description of heart/ cardiovascular late effects for more information.

- Yearly mammograms and examination by breast cancer specialist.
- Consideration of physical/ occupational therapy for arm pain, weakness, or swelling.
- Rapid evaluation for new arm swelling, redness, or pain, especially with fever.

Follow-up Care

Breast Cancer

After receiving treatment for breast cancer, it is important for survivors to adhere to their physician's plan for Follow-Up Care. Guidelines developed by the National Comprehensive Cancer Network state that survivors who have had breast conserving therapy (lumpectomy) should have their first mammogram approximately 6 months after completing radiation therapy, then annually. Survivors who underwent mastectomy should have a mammogram annually. In addition, breast MRI may be considered for survivors with the BRCA 1 or 2 genes. Survivors should be seen by their oncologist every 4 to 6 months for the first 5 years and then annually. Women who are taking tamoxifen should be seen annually by a gynecologist and be sure to report any vaginal bleeding to their physician right away.

Many survivors find it difficult to not have periodic CT scans or bone scans to look for spread of the cancer. Research has shown that the type of treatment, response to treatment, and overall survival are all the same, regardless of when the treatment is initiated. In other words, the outcomes are similar for those who are treated for metastases found on routine screening (with no symptoms present) and women who are not treated until those metastases cause symptoms. Therefore, we no longer screen patients without symptoms for metastases, but rather wait until symptoms develop.

- Mammogram annually (first one six months after therapy for survivors who had lumpectomy and radiation therapy).
- Seen by oncologist every 4-6 months for 5 years, then annually.
- Women taking tamoxifen should see a gynecologist annually and notify their physician of any vaginal bleeding.

Please take a moment to fill out the Oncolife Survey.

© Trustees of the University of Pennsylvania
